# A Protocol Guide to Micro Milling for Bio-Microfluidics

**DOI:** 10.21769/BioProtoc.5459

**Published:** 2025-10-05

**Authors:** Hannah L. Viola, Vishwa Vasani, Shuichi Takayama

**Affiliations:** 1 The Parker H. Petit Institute for Bioengineering and Bioscience, Georgia Institute of Technology, Atlanta, GA, USA; 2 School of Chemical & Biomolecular Engineering, Georgia Institute of Technology, Atlanta, GA, USA; 3 The George W. Woodruff School of Mechanical Engineering, Georgia Institute of Technology, Atlanta, GA, USA; 4 Wallace H. Coulter Department of Biomedical Engineering, Georgia Institute of Technology and Emory School of Medicine, Atlanta, GA, USA

**Keywords:** Microfluidics, Organ-on-a-chip, CNC machining, Rapid prototyping, Micro mill, PDMS casting

## Abstract

Micro milling is a subtractive manufacturing method for fabricating micro-scale three-dimensional features from hard substrates like acrylic, wood, or metal. It enables rapid prototyping of biomicrofluidic devices and master molds, offering advantages over traditional fabrication methods like photolithography. Micro milling is seldom applied in the fabrication of organs-on-a-chip, in part due to its requirement for knowledge of computer numerical machining techniques that are required to program and operate micro mills. This protocol provides practical guidelines for micro milling–based fabrication of organs-on-a-chip, including toolpath optimization, SolidWorks and Fusion workflows, and troubleshooting tips. A case study demonstrates the design and fabrication of master molds for a human airway-on-a-chip, validated in a recent publication. This resource supports the expansion of micro milling techniques into organs-on-a-chip, which will enhance capacity for rapid device prototyping and design of more complex 3D features that better recapitulate human physiology.

Key features

• Stepwise guide to lung-on-a-chip design and fabrication via micro milling, a specialized type of computer numerical control (CNC) machining.

• Demonstration of model design, tool path optimization, micro milling, device assembly, and cell culture.

• Example SolidWorks and Fusion documents illustrating best practices for model design and toolpath generation.

## Background

Rapid micromachine prototyping helps optimize biomicrofluidic devices to culture organoids, generate particles, and create organs-on-a-chip. Environments such as university or community-led “makerspaces” have expanded access to micromachining equipment, such as computer numerical control (CNC) machines and 3D printers, for rapid prototyping. Micro milling, in particular, is well suited to microfluidics for biomedical applications (i.e., biomicrofluidics) [1]. This technique is inexpensive, provided that the capital investment in a micro mill has been met, and is rapid and flexible in its capabilities. Micro mills are CNC machines that execute a computer-designed path to make precision cuts via subtractive manufacturing. Micro mills differ from traditional CNC mills in that they operate at the millimeter-to-submicron scale, whereas traditional CNC machining centers work at a macroscopic scale. Therefore, micro milling has been utilized to fabricate microfluidic devices, including those with biomedical applications where rapid prototyping is desirable [2]. However, sparse resources exist to introduce micro milling technology to biomedical researchers who might benefit from its advantages in biofabrication. Here, we provide a step-by-step protocol describing lung-on-a-chip microfluidic device design, fabrication, and cell culture. Techniques described herein are broadly applicable to organ-on-a-chip design and fabrication with micro mill technology.

Micro milling is advantageous for the fabrication of certain microscale features in a relatively quick and low-cost fashion compared to other manufacturing methods. Compared to traditional photolithography, micro mills can more readily fabricate three-dimensional curved shapes and handle low width-to-height aspect ratio features. Additionally, traditional photolithography-coupled soft lithography is limited to ~100–200 μm depth and two-dimensional features [2]. Micro milling eliminates the need to generate a lithography mask for each new design, which lowers the number of fabrication steps required for quicker iteration. Furthermore, CNC-based micro milling needs little supervision once set up and does not require a cleanroom. However, micro milling is disadvantageous at the lower nanoscale compared to photolithography. Additionally, micro milling cannot necessarily perform undercuts and has limitations to the radius of curvature that can be achieved on inside corners. Finally, micro-milled surfaces tend to have a greater surface roughness than comparable surfaces created by photolithography-coupled soft lithography, which could affect cell viability [2] and surface adhesiveness.

**Figure 1.**
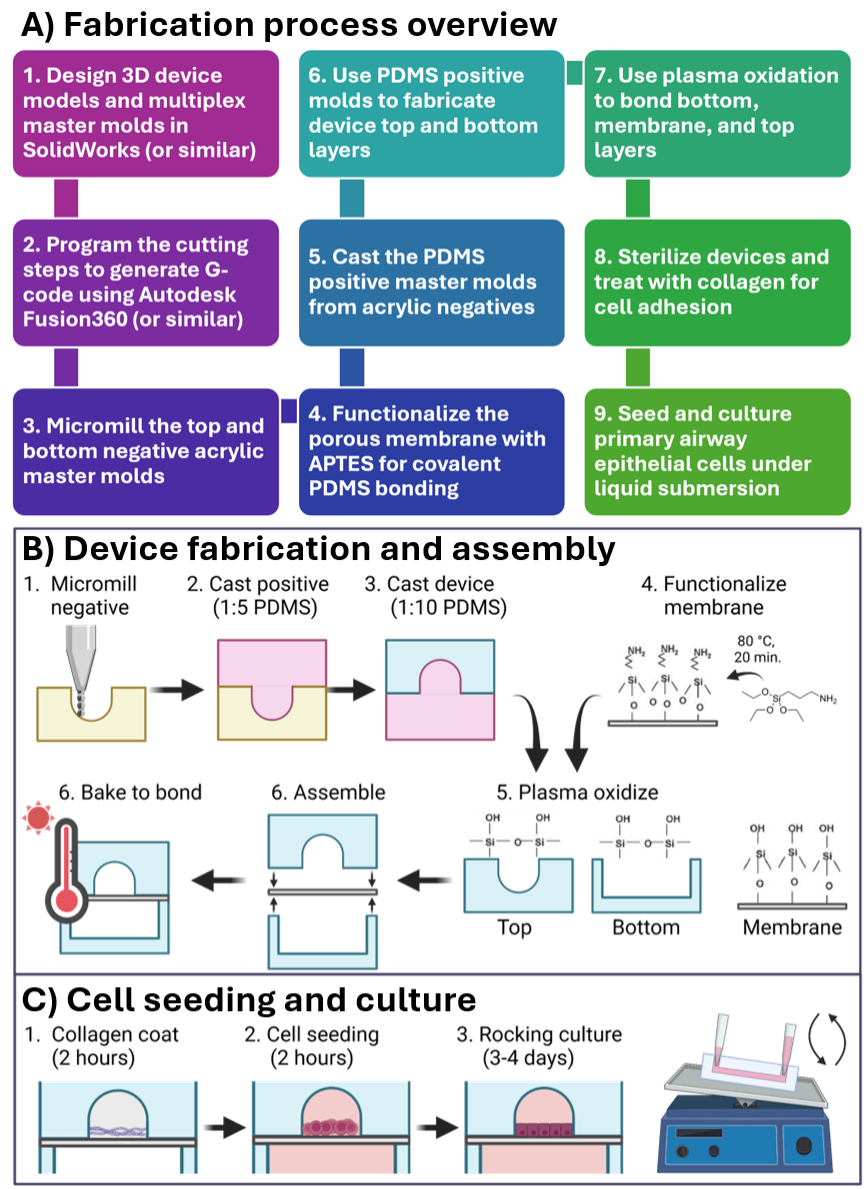
Protocol steps described herein. (A) An overview of the fabrication steps for a micro-milled organ-on-a-chip that are described in this protocol. (B, C) Illustrations of protocol steps as published in Viola [8] Lab on a Chip. This figure is reproduced from our article in Lab on a Chip available at https://pubs.rsc.org/en/content/articlelanding/2024/lc/d3lc00957b. This figure is reprinted under Clause 3.2 of the Copyright Owner(s)’ Rights under the Royal Society of Chemistry’s License to Publish Agreement.

In comparison to 3D printing, micro milling is compatible with harder substrates that are difficult to extrude, such as wood, ceramic, and metal, for durable master mold generation. Additionally, a direct comparison of micro milling of acrylic vs. 3D printing of UV-curable substrate to fabricate the same features found that micro-milled square channels had a smoother surface finish and more defined side walls with greater feature accuracy [3]. Additionally, z-axis resolution is limited in most 3D printing situations, so that at the microscale, noticeable ridging can occur on curved surfaces [4]. Micro mills are uniquely suited to fabricate curved features without this artifact. However, for larger-scale (micron to centimeter) designs requiring bioprinting, overhangs, or channels passing over or under one another, 3D printing is most appropriate [5,6]. Polymer parts are also fabricated by injection molding, which has demonstrated precision and desirable reproducibility [7]. However, for pilot studies that are typical of rapid prototyping applications, micro milling is substantially more economical, as injection molding requires a costly start-up investment in custom molds that are designed for repeated long-term use. Alternatively to machining, microscale features are also fabricated by laser techniques and piezoelectric rather than mechanical mills; however, the aforementioned accessibility and ubiquity of milling equipment makes it desirable in microfluidic prototyping, especially for the creation of a master mold that can be cast to create poly(dimethylsiloxane) (PDMS) devices (Figure 1) or for directly making final parts out of hard plastics when the microfluidic systems deal with small sample sizes such as in scRNAseq-on-a-chip or PCR-on-a-chip to prevent absorption of analytes into PDMS. The overall balance of micro milling’s advantages vs. limitations should be taken into account in the determination of its appropriateness for a particular application. For examples of the application of micro milling in biology, we document several recent works and their milling parameters, as available, in Table 1 below.

**Table 1.** Example biomicrofluidics and manufacturing parameters

Citation	Material	Application	End mill (s)	Spindle speed	Feed rate	Depth of cut/ stepdown	Manual stepover
[8] Viola 2024	**Acrylic**	**Nanoinjector**	**500 μm flat** **200 μm ball**	**10,000 rpm** **30,000 rpm**	**25.53 mm/min** **12.27 mm/min**	**0.25 mm** **0.10 mm**	**0.125 mm** **0.050 mm**
[9]	Acrylic	Bladder on a chip	3 μm carbide flat	Not reported	Not reported	Not reported	Not reported
[10]	Poly(methyl methacrylate) (PMMA)	Microfluidic vascular model with integrated electrodes	254 μm	Not reported	Not reported	175 μm	Not reported
[11]	PMMA	Tumor spheroid device	1 mm carbide square 2-flute	18,000 rpm	60 mm/min	100 μm	Not reported
[12]	Polystyrene	Macrophage culture on chip	Not reported	Not reported	Not reported	Not reported	Not reported
[13]	Aluminum	Spiral-shaped test device	200 μm	10,000–20,000 rpm	50–150 mm/min	5 μm	Not reported
[14]	PMMA	Test device with curved channels	350 μm 2 flute steel	4,000 rpm	10 mm/min	0.5 mm	Not reported
[15]	Brass	DNA gel electrophoresis	500 μm carbide 200 μm carbide 100 μm carbide 50 μm carbide	40,000 rpm	200 mm/min 100–150 mm/min 50–75 mm/min 10–20 mm/min	Not reported	Not reported

## Materials and reagents

**Biological materials**

1. SAEC, human small airway epithelial cells (Lonza, catalog number: CC-2547)

**Reagents**

1. SAGM^TM^ Small Airway Epithelial Cell Growth Medium BulletKit^®^ (Lonza, catalog number: CC-3118)

2. ReagentPack^TM^ subculture reagents, 100 mL (Lonza, catalog number: CC-5034)

3. Corning^®^ Collagen I, rat tail, 100 mg (Corning, catalog number: 354236)

4. (3-Aminopropyl) triethoxysilane (APTES) (Millipore Sigma, catalog number: 440140-100ML)

5. Dowsil Slygard 184 Silicone Elastomer kit 0.5 Kg (1.1 lb) clear (Dow Chemical, Krayden, Manufacturer: dc4019862; Vendor: 4019862)

6. UltraPure^TM^ DNase/RNase-free distilled water (or equivalent) (ThermoFisher, catalog number: 10977015)

7. 1 M sodium hydroxide (Sigma Aldrich, CAS number: 1310-73-2)

8. DPBS, no calcium, no magnesium (ThermoFisher Scientific, Gibco, catalog number: 14190250)

**Laboratory supplies**

1. 24 in. × 48 in. × 0.250 in. clear acrylic sheet (4 per pack) (Home Depot, PlexiGlass, model: MC2448250)

2. Corning^TM^ Transwell^TM^ multiple well plate with permeable polycarbonate membrane inserts, 0.4 μm pore size, for 6-well plates (Fisher Scientific, catalog number: 07-200-165, Corning^TM^, catalog number: 3412)

3. 1,000 μL pipette tips, no filter, sterile (ThermoFisher, catalog number: 9401113)

4. 200 μL pipette tips, with filter, sterile (Corning Millipore Sigma, catalog number: CLS4823-960EA)

5. Nunc^TM^ Square BioAssay dish, 25 mm non-treated (ThermoFisher, catalog number: 240835)

6. Single edge razor blades (ULINE, catalog number: H-595B)

7. Cole-Parmer low-form beaker, glass, griffin style, graduated, 400 mL; 8/Pk (Cole-Parmer, model: UX-34502-45)

8. USA Lab rare earth magnet pill-shaped stir bars, various sizes (USALAB, SKU: REP-8x22)

9. Labeling tape, white, W × L 3/4 in. × 500 in. (Sigma-Aldrich, catalog number: Z768294)

10. Extra Fine Graefe forceps (Fine Science Tools, catalog number: 11150-10)

11. 10 mL/cc syringes with 18Ga needles and caps, disposable syringe, single sterile individually packaged (20Pack-10ML) (Premium Vials, SKU: B07DVXPNKT)

12. Fisherbrand^TM^ high precision metal scalpels (Fisher Scientific, catalog number: 08-920B)

13. Self-healing cutting mat, 24 × 18", blue (ULINE, catalog number: S-18545)

14. Grip-N^TM^ hot mill gloves (ULINE, catalog number: S-19220)

15. Aluminum foil roll, heavy-duty, 24" × 500' (ULINE, catalog number: S-22910)

16. 1.88 in. × 54.6 yds. Heavy-duty shipping packaging tape with dispenser (The Home Depot, Scotch Brand, #100149185, model: 3850-RD-DC, Store SKU #545597)

17. 3 M 667 Scotch^®^ double-sided tape with dispenser, 3/4" ×11 yds (ULINE, Scotch Brand, catalog number: S-18907)

18. Integra Lifesciences disposable standard biopsy punches, disposable biopsy punch with plunger, 1.5 mm, 33-31A-P/25 (Grayline Medical, by Integra Lifesciences Corp, SKU: 33-31A-P/25)

19. MilliporeSigma^TM^ Steriflip^TM^ sterile disposable vacuum filter units, 0.22 μm (Fisher Scientific, catalog number: SCGP00525)

20. Snap-seal disposable plastic sample containers (Corning^TM^, catalog number: 1730-10)

## Equipment

1. Computer (PC) running up-to-date Windows operating system (any)

2. Micro mill (CNC Mini-Mill/GX, Minitech, catalog number: CNC Mini-Mill/GX)

3. Shop vacuum (12 Gallon 5.0 Peak HP NXT wet/dry shop vacuum with filter, locking hose and accessories (The Home Depot, catalog number: 1002916304)

4. Pressurized air source (ALPHAGAZ^TM^ 1 Grade Nitrogen, Size 300 High Pressure Steel Cylinder, CGA 580) (Airgas, catalog number: NI AZ1300SMT)

5. Gram scale (Uline Balance Scale, ULINE, catalog number: H-9884)

6. Orbital mixer (Orbital mixer, FlackTek, Inc., catalog number: Speedmixer DAC 150.1 FVZ-K)

7. Vacuum desiccator (Nalgene^TM^ Transparent Polycarbonate Classic Design Desiccator, ThermoFisher, catalog number: 5311-0250PK)

8. Oven (65 °C) (Heratherm General Protocol Oven, ThermoFisher, catalog number: 51028112)

9. Oven (120 °C) (Heratherm General Protocol Oven, ThermoFisher, catalog number: 51028112)

10. 500 μm square carbide end mill (TR SERIES 2 FLUTE MICRO END MILLS ≤ 0.0600, Performance Micro Tool, catalog number: TR-2-0200-S)

11. 100 μm ball nose carbide end mill (TR SERIES 2 FLUTE MICRO BALL END MILL, Performance Micro Tool, catalog number: TR-2-0040-BN)

12. Plasma oxidizer (Tergeo plasma cleaner, Pic Scientific)

13. Certified chemical fume hood (60″ Lab Fume Hood Vertical Sash, CleaTech, catalog number: SKU 1100-3-B)

14. Hotplate with magnetic stirring (USA Lab Hotplate Stirrer 380, 5L, TUV Certified, USALAB, catalog number: SKU: USA-H380-PRO)

15. Laser thermometer (REED INSTRUMENTS Infrared Thermometer: R2300, Max Temp/Min Temp, Adj @ 0.10 to 1.00, Full Size Body, Grainger, catalog number: R2300)

16. Pipette aid (Drummond DP-101 Portable Pipet Aid XP Pipette Controller, 110V, Pipette.com, SKU: DP-101)

17. pH probe (Ohaus ST2200-F Benchtop pH Meter Kit, Hogentogler, catalog number: SKU ST2200-F)

18. Refrigerated tabletop centrifuge (Sorvall^TM^ ST 16 Centrifuge Series, ThermoFisher, catalog number: 75004380)

19. Inverted brightfield microscope (DMi1 Inverted Microscope, Leica Microsystems, catalog number: 11526231)

20. Band saw (JET JT9-708115K Model JWBS-14CS 1HP 1-Phase 115/230V 14" Closed Stand Bandsaw, Global Industrial, Model: WBB57825)

21. Cell culture incubator (BB15 CO2 Incubator, ThermoFisher, catalog number: 51023121)

22. Hemocytometer or cell counter (Cellometer Auto T4 Bright Field Cell Counter, Revvity, catalog number: CMT-AT4P)

23. Vortexer (Original Vortex Genie 2, Pipette.com, catalog number: 00-SI-0236)

24. Micropipettes (Gilson PIPETMAN Neo Pipette Set, Gilson, catalog number: GNPST)

25. LN2 tank (Locator 6 Rack and Box System, ThermoFisher, catalog number: CY50985)

26. BSC class II (1300 Series Class II, Type A2 Biological Safety Cabinet Packages, 120 V 50/60 Hz, ThermoFisher, catalog number: 1323TS)

## Software and datasets

1. SolidWorks 2025 by SolidWorks (https://www.solidworks.com/)

2. Mach Mach3 3.043.066 by ArtSoft (https://www.machsupport.com/software/mach3/)

3. Fusion v.2.0.19941 by AutoDesk (https://www.autodesk.com/products/fusion-360/overview)

## Procedure

**Part I: Design and fabrication of master molds**

**Before you begin**: Microfluidics for biological applications have unique design requirements to accommodate the culture and manipulation of cells. Most prominently, they should be biocompatible (non-cytotoxic) while minimally absorbing or adsorbing components of cell culture medium. They should also be oxygen and carbon dioxide–permeable for appropriate cellular respiration and optically transparent for online monitoring of cell phenotypes and behavior. Finally, the material needs to be amenable to bonding with both itself and other materials, such as poly(ethylene terephthalate) (PET), to form closed channels and multilayered devices. These requirements are all met by the most common material used for biomicrofluidics, PDMS. However, it is not recommended to micro-mill PDMS because it is too soft. Instead, a master mold can be created in a hard substrate that does not bond with PDMS, from which PDMS devices can be cast. A master mold-based technique, rather than direct milling of each device, has the advantage of reducing the amount of milling required and improving the reproducibility of cast devices.

**Figure 2.**
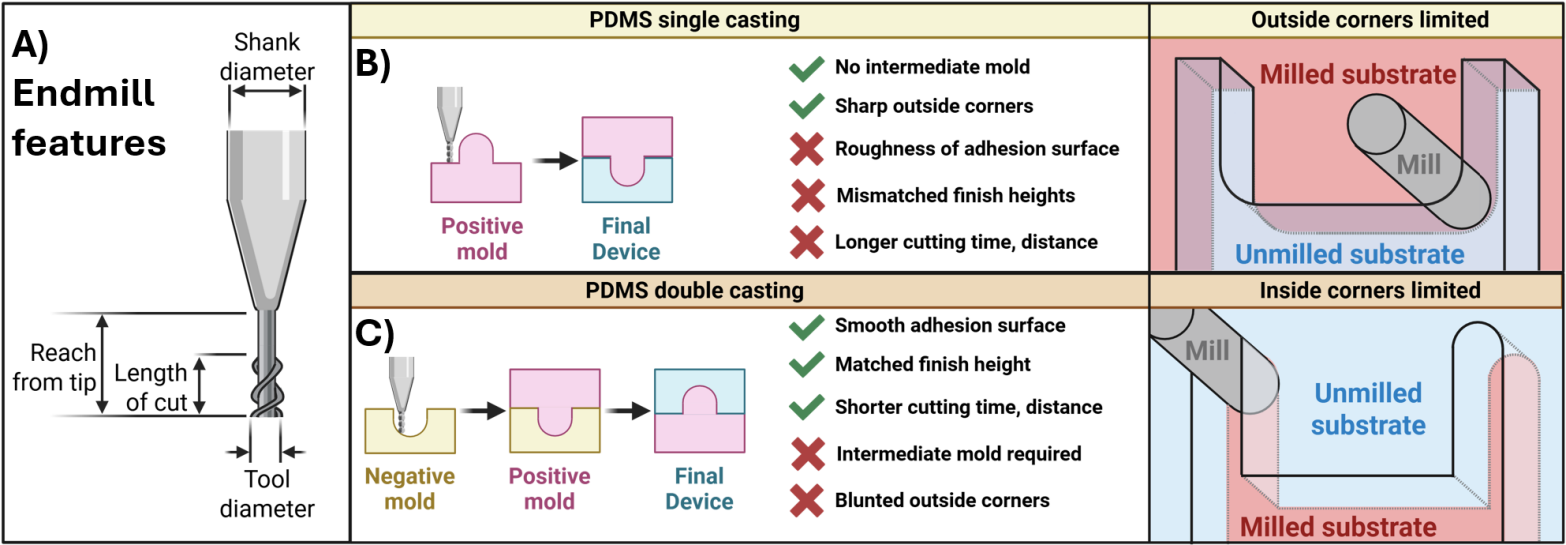
End mill features and casting methods. (A) Parameters for a typical end mill that are required to enter into path-generating algorithms, such as Fusion (AutoDesk). (B,C) Comparison of single- and double-casting methods for microfluidic devices fabricated from micro-milled master molds. (B) Single-cast master molds allow sharp outside corners with radius limitations for inside corners. (C) Milling the negative mold provides the inverse advantages.

*Note: Example SolidWorks files are provided in supplementary material for the device featured in this protocol. Steps are described for general model creation; for more detailed information regarding the use of SolidWorks software, see* *https://help.solidworks.com/* .

To cast a device directly from a micro-milled surface, the positive or negative device can be fabricated by the mill. Surface roughness imparted by milling can negatively impact the adhesion of PDMS-casted device parts [2]. Therefore, an alternative strategy is to mill the device features as negatives directly into a smooth naïve surface, such as acrylic plastic (Figure 2). The naïve surface maintains a low surface roughness that facilitates adhesion. The positive mold is then cast in PDMS with a high concentration of crosslinker. This results in a PDMS-positive mold that does not bond to the PDMS device cast and can be separated easily after baking. This method works even without surface functionalization of the positive PDMS mold (unpublished observation). As an added benefit, milling negative rather than positive features has been shown to achieve smaller tolerance, greater accuracy, and smaller feature size than positive feature milling and avoids milling-inherent limitations to inside corner radius [15,16].

**A. Create top and bottom mold models in SolidWorks**

1. Create a folder to store all SolidWorks documents for this project. After beginning work, do not move this file or rename it or its components. This ensures that file references are not interrupted, which may break assemblies.

**Caution:** Do NOT rename files from the File Explorer. If new file names are required, utilize the “Save As” function in SolidWorks and select the option to move all file references to the new document.

2. Create the top channel of the device as a SolidWorks Part document (example file found in Supplementary Material: “Final Top.SLDPRT”). Save this part to your SolidWorks folder.

3. Open the Top part. Create a reference plane called “X reference” that is offset 0 mm from the Top plane. Repeat for the Right and Front planes and name these “Y reference” and “Z reference,” respectively. These reference planes will be the foundational planes used to define any additional reference geometry. Defining reference geometry relative to these planes ensures that features can be translocated in space by moving only the reference planes without breaking other parts or features, which are also linked to the reference geometry.

**Figure 3.**
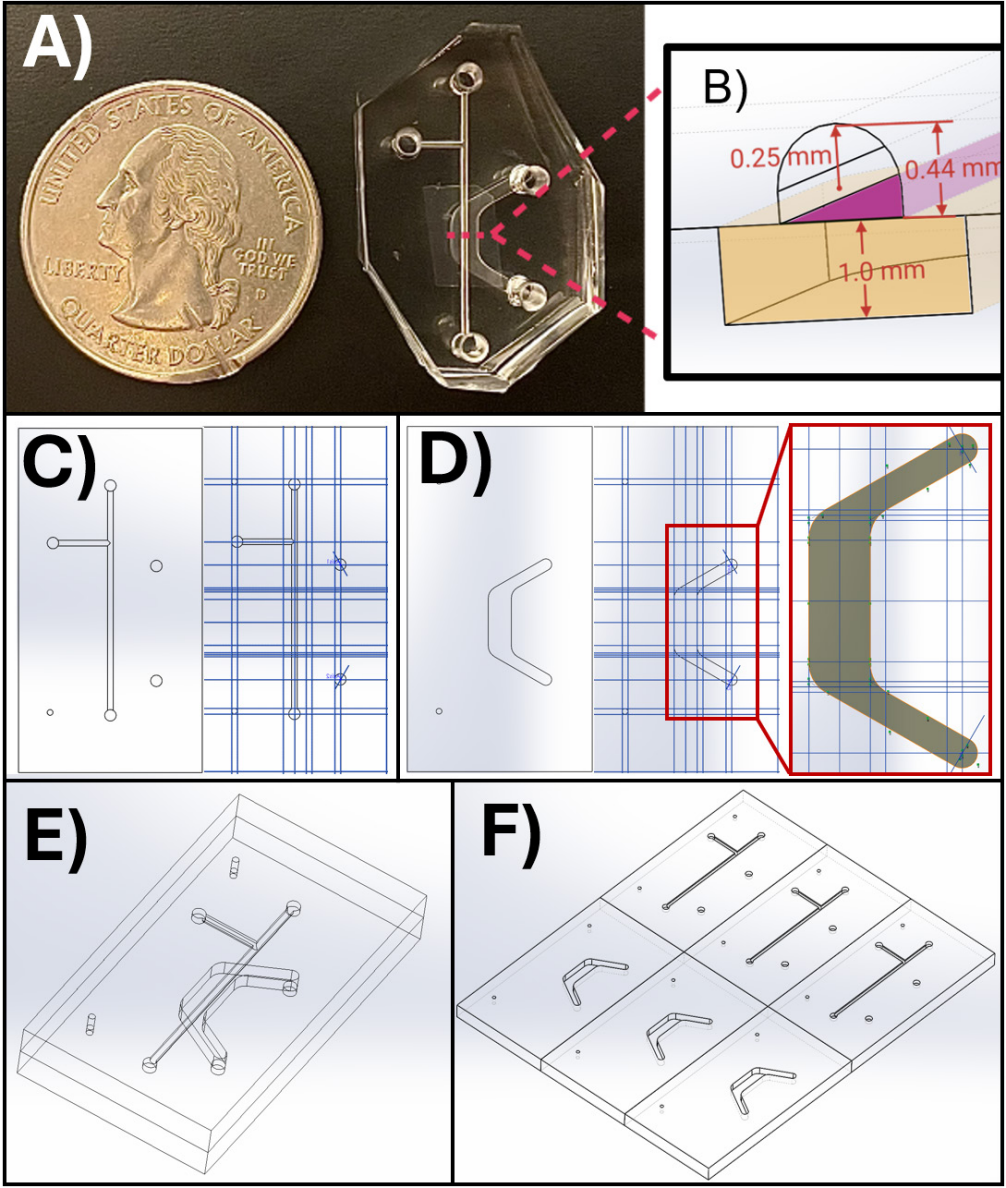
SolidWorks models for the microfluidic device channels and master mold. (A) The microfluidic device appears next to a standard United States quarter for scale, with key dimensions illustrated in a cross-section view in (B). (C, D) SolidWorks models showing the device design and reference planes (blue lines) for the top (C) and bottom (D) channels. (E) Assembled microfluidic device. (F) Master mold design for micro milling into acrylic. The top panel appears as published in Viola [8], Lab on a Chip. This figure is reproduced from our article available at https://pubs.rsc.org/en/content/articlelanding/2024/lc/d3lc00957b. This figure is reprinted under Clause 3.2 of the Copyright Owner(s)’ Rights under the Royal Society of Chemistry’s License to Publish Agreement.

*Note: We strongly recommend using relative reference geometry, such as planes, axes, and points, to define sketch locations and dimensions. We have found it useful to define planes relative to a “master” reference plane that can be moved so that features can be translocated in space without disturbing relationships between other planes and sketches. Therefore, one may create master X, Y, and Z reference planes offset 0 mm from the Front, Top, and Right planes, respectively. The remainder of the reference planes are defined relative to these master references. Then, features that refer to the master references and their derivatives can be moved without breaking mates between sketch components and reference geometry. It is not recommended to define sketch planes relative to object surfaces, because changes in the object may break the sketch or plane mates. Example reference plane geometry is displayed in Figure 3.*

4. Create a new sketch on the Front plane and draw a rectangle with one of the corners mated to the intersection of the Front, Top, and Right reference planes (the origin). This will anchor the part to the origin. These mates can later be broken if needed, but it is useful to keep your drawing in a defined location unless moving it is necessary.

5. Exit the rectangle sketch. Boss-extrude the rectangle to create a box. This box represents the substrate comprising the top (or bottom) channel of the device.

6. On the Z reference plane, create a new sketch. Draw relevant two-dimensional features on this plane. This includes entry and exit holes and flat features such as rectangular channels. All 2D features may be located in the same sketch as long as their depth into the material is identical.

*Note: In SolidWorks, a fully defined sketch element (e.g., a line or circle) will appear black, whereas an underdefined element will appear blue. A fully defined sketch can only satisfy its specified geometric relationships with one conformation. For example, for two points with defined locations, a straight line that must intersect both points can only exist in one spatial orientation. Fully defining sketch entities is important to control the behavior of sketch elements.*

7. To create features from the Z plane drawing, execute an extruded cut from the sketch.

8. To create 3D features such as the curved channels in the example device, create a new reference plane that is offset from the X or Y plane. Create extruded cuts (or other more complex features) across the stock material.

9. When the top part is complete, save it. Then, save the completed part with a new name (example file “Final Base.SLDPRT”). This will become the opposite channel. Starting the new channel from the completed part carries over the reference geometry and features created for the completed channel, which makes it easier to design the complementary channel so that it matches the already completed features.

10. Complete the bottom channel in a similar fashion to the top, deleting top channel features as necessary. Include guide markers (small circles shown on the left side in Figure 3E) to enable proper top and bottom channel alignment during device assembly. Additionally, if necessary, return to the top channel and add markers for the entry and exit points of the bottom channel (larger circles shown on the right in Figure 3C). It is difficult to determine where to punch these holes during fabrication without an indicator. However, if one is not included, one may use a marker to denote the location of exit holes (see Figure 7E).

11. When the top and bottom parts are complete, create an assembly file containing both parts and align and mate the parts to ensure they fit together correctly. The finished device model assembly is shown in Figure 3E.

**Caution:** It is not recommended to define reference geometry in an assembly file. It is difficult to properly reference sketch geometry to planes in an Assembly file. Changes to each part should be executed within individual part files.

12. When the parts are properly created and look correct in a device assembly, make another assembly to create the acrylic master mold. Import multiple copies of each top and bottom part and align them as shown in Figure 3F by creating mating relationships to line up multiple top or bottom parts into a contiguous assembly. This file is imported to Fusion for the generation of micro mill tool paths to create an acrylic master mold that fabricates three devices.

*Note: Part copies within assemblies retain all properties of the original part; that is, if the “Top.SLDPRT” file is edited, these changes are reflected in every copy present in the assembly file.*

**B. Design tool paths in AutoDesk Fusion**

*Note: Device design can be performed in Fusion directly, and toolpath generation can be accomplished in SolidWorks, but we have found that SolidWorks performs better at 3D modeling applications, while Fusion is more suited for toolpath generation.*

**Before you begin:** CNC machining requires optimized parameters for the tool path to ensure faithful cuts and minimize tool wear. Tool paths refer to the end mill’s feed rate, spindle speed, and tool size, in addition to the trajectory of the end mill. These parameters must be custom-generated based on the device’s geometry, the material being machined, and tool requirements (i.e., tool size, end shape, number of flutes, etc.). Software such as AutoDesk Fusion is available to semi-automatically generate such custom tool trajectories based on inputted feed and speed parameters. We provide a custom tool path generated in Fusion and its accompanying SolidWorks design file as an example. Not only does the proper path ensure that end mills last longer, but it also prevents overheating of the substrate. Importantly, plastic and acrylic materials that are typically utilized for rapid prototyping can melt easily due to an overheated end mill. In turn, melted chips can obstruct the tool’s flutes and inhibit proper cutting, exacerbating tool wear and heating. This can culminate in tool breakage. For these reasons, both optimal path settings and lubrication are critical. Lubricant can be added to the surface of the substrate and can be periodically reapplied during the cut. Additionally, a focused air jet removes chips from the cutting area.

The most important parameters to generate a tool path are feed rate, spindle speed, depth of cut, and stepover (colloquially referred to as “feed ‘n speed”). These depend on the type of cut being performed. General design principles consist of rules of thumb that are adapted from macroscale CNC machining, with some modifications. First, the feed rate depends on the maximum spindle speed and the tool size. Smaller tools require faster spindle speeds and slower cutting rates. In general, larger tools can succeed with lower spindle speeds and faster feed rates. Unoptimized spindle speeds and cutting rates are more likely to overheat or break the end mill. Unoptimized cuts are especially likely to cause problems with plastic substrates that are prone to burring and melting. Therefore, precise values need to be chosen to avoid melting chips that clog the end mill flutes and lead to tool breakage. The precise values can be calculated by quantifying the chip load. Chip load is the theoretical amount of material that is fed into each cutting edge with each rotation of the spindle. It is calculated by relating feed rate, spindle speed, and cutting edges (e.g., a 2-flute end mill has two cutting surfaces) [15]:

$$Chip\;load\;per\;tool=\;\frac{Feed\;rate\;\left(\frac{inches}{min}\right)}{Spindle\;speed\left(\frac{number\;of\;rotations}{min}\right)\times \#\;Cutting\;edges}\;\;\;\;\;\;\;\;\;\;\;\;\;\;\;\;\;\;\;\;(1)$$

An example spreadsheet to calculate feed and speed parameters for a micro-milled part is available in Supplemental Materials (“MicroMill Feed & Speed Calculations.xlsx”). Chip load should remain between 2% and 4% of the tool diameter for most situations. A lower chip load may be required for harder materials, and a higher chip load for softer materials. Permissible chip load limits will also depend on the melting temperature of the substrate, with melting-prone materials such as plastics requiring a lower chip load. Finally, feed and speed rates depend on the particular cut being made and the tool quality. Holes, angled cuts, and corners require slower speeds, with a rule of thumb that these use around 50% of the flat-surface feed rate. Additional parameters to tune include manual and maximum stepover and depth of cut. Stepover is an important parameter that defines how much the tool will overlap with the previous adjacent cut. A smaller stepover will produce a smoother surface finish, but will take longer to cut and will wear out the tool more. We provide some general starting point guidelines for these parameters in Tables 2 and 3. Note that the values provided here are starting points for an acrylic substrate, and exact numbers should be optimized by the user for their particular situation, as described above.

**Table 2.** Starting point values for feed and speed parameters

Parameter	Feed rate	Spindle speed
Flat cuts (base)^1^	100% of base	100% of base
Corner or angle	50% of base	25% of base
Boring (holes)	50% of base	25% of base

^1^Base feed rate and spindle speed are those used for an unobstructed flat surface and require optimization for the end mill and material used.

**Table 3.** Starting point values for more feed and speed parameters

Parameter	Value
Manual stepover	50%–90% of tool diameter
Maximum stepover	95% of tool diameter and < length of cut
Maximum depth of cut/stepdown	20%–50% of tool diameter
Chip load	2%–4% of tool diameter (depends on stock material)

*Note: An example Fusion file is provided in the Supplementary material for the device featured in this protocol. Steps are described for general simulation creation, but for more detailed information regarding the use of Fusion software, see* *https://www.autodesk.com/learn/ondemand/collection/self-paced-learning-for-fusion* .

1. Import the master mold SolidWorks assembly to Fusion.

a. Ensure the box in the top left corner says “MANUFACTURE.”

b. Click the top-left corner icon that consists of a square made up of larger squares (“Data Panel”).

c. Click *New Project* (top-right button in data panel) and name your project.

d. When the project appears on the data panel, double-click to enter the project.

e. Click *Upload* on the top-right of the data panel. Import the 6-plex assembly part as well as the SolidWorks part file it was built from. If you try to upload the assembly without the part file, Fusion will complain.

f. When the file is uploaded, click and drag it from the data panel onto the workspace grid. The assembly should now appear in the grid. Ensure that the top left box still says “MANUFACTURE” and not “DESIGN”.

2. Enter the tool settings for your end mills.

a. Click the Tool icon under the *Manage* panel at the top of the screen.

b. The tool library menu appears. Under *User libraries* > *Documents*, select *Untitled* or create another tool library if you like.

c. Once a library is selected, the plus sign on the top left will become un-grayed out. Click the plus sign to add a tool.

*Note: While Fusion already has a library of end mills, micro mills are not usually represented in this library, so tools must be entered manually.*

3. On the *Tool* tab, create two new tools for the 500 and 200 μm end mills.

a. Input key tool properties for each tool, including end mill diameter and shape (i.e., ball vs. square) and number of flutes. Not every end mill property is necessary to define a cut, although extra information can be useful for Fusion to optimize the toolpath.

b. Not all information is usually available for entry into the software. The end mill material is carbide. The number of flutes, overall length, and flute length (i.e., length of cut) are available from the manufacturers. The length below holder and shoulder length are not always reported for micro mills, but a value is required, so enter a number higher than the flute length but lower than the overall length. Fusion uses this information to detect potential collisions of the tool with the substrate, but lacking this information does not generally impact path generation unless extremely complex features are required, in which case this information might be obtainable by contacting the end mill’s manufacturer.

c. Change the tab to *Cutting data* and set the RPM for the tool. Fusion will automatically calculate the recommended surface speed for this RPM and tool diameter. These estimates are very close to our calculations (“Micro mill Feed & Speed Calculations.xlsx”, Supplemental Materials). Scroll down to the *Feedrates* panel. Enter the cutting feed rate that is calculated by a chip load of 4% (see “Micro mill Feed & Speed Calculations.xlsx”, Supplementary Materials). Fusion’s automatically calculated feed rate is too high for micro mill applications. Manually enter the values calculated by the feed and speed spreadsheet here.

d. Scroll down and change coolant to *Disabled*. We have found that including coolant in the simulation causes errors in running the G-Code for unclear reasons.

e. Repeat sub-steps B3a–d for the 200 μm end mill. Ensure that under the *Post processor* tab, the 500 μm end mill is indexed first (Number, Length offset, Diameter offset should be set to 1). Check the *Manual tool change* and uncheck *Live tool*. For the 200 μm end mill, repeat this but with the index set to 2. This is done automatically if the tools are entered in the order that they will be used in the cutting path.

**Figure 4.**
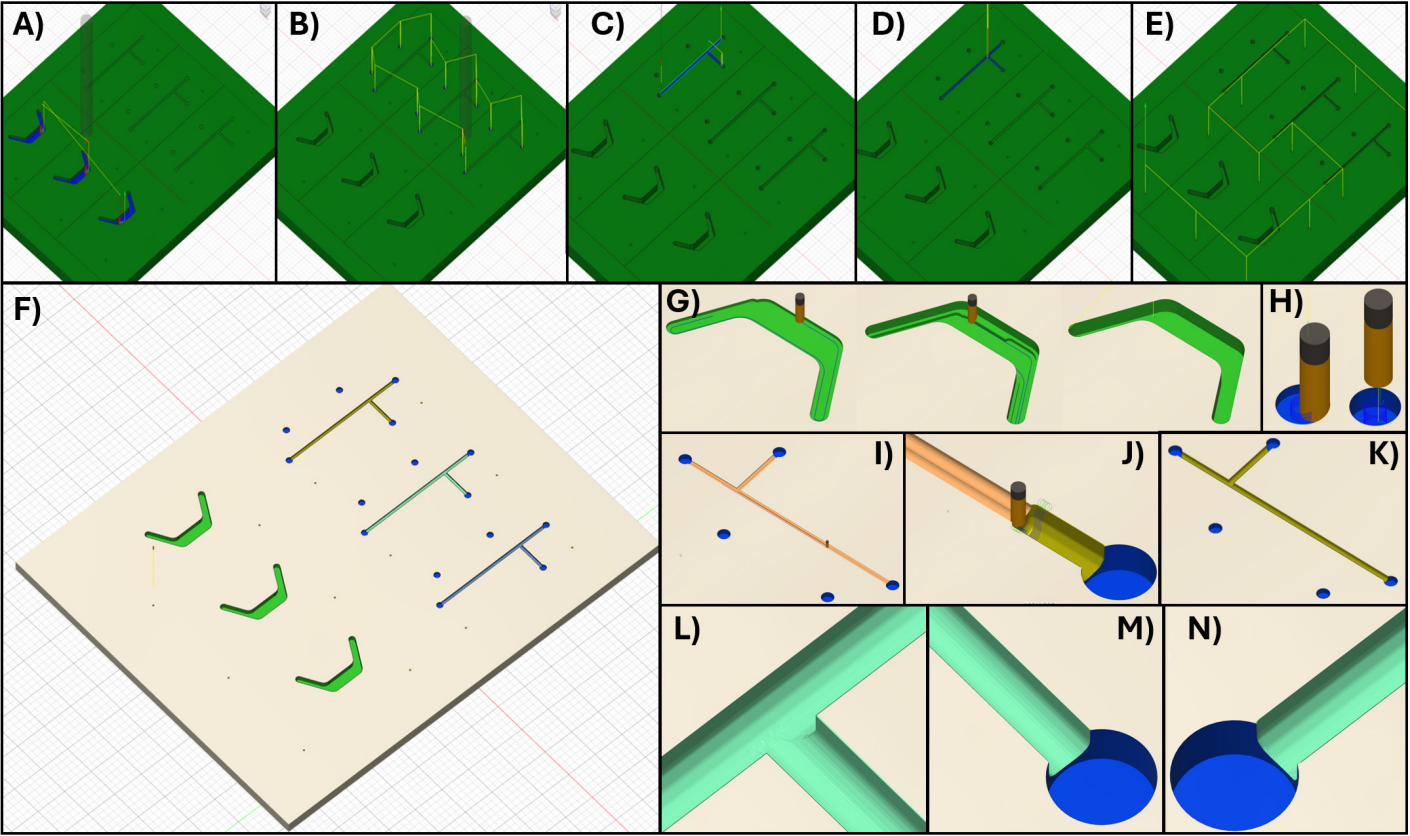
Fusion simulations. (A) The first step in Fusion uses a 500 μm flat tool to pocket the bottom channel. (B) Then, with the same tool, the 0.5 mm diameter holes are bored. (C) Next, switching to the 200 μm ball-nose end mill, the top channel is roughed out with a low stepover to clear material. (D) Next, a low stepdown, low-stepover parallel facing step is applied to the top channel to create a smooth finish. (E) Finally, the marker holes are bored. (F) The milled part is generated by the Fusion simulation. (G) Images of the bottom part during pocketing. (H) Simulation of boring the 0.5 mm entry/exit holes. (I) Rough pocketing of the top channel. (J) Low-stepover parallel finishing step for the top channel. (K) Fusion simulation of the milled top channel. (L, M, N) Close-up images of the simulated top channel, showing minor ridging but a smooth surface overall and cuts faithful to the model design.

4. Define the stock in Fusion.

5. Set the Origin at the lower-left corner and top face of the Stock.

6. Program the end mill cuts. Usually, a clearing step with a larger tool is appropriate to remove material in preparation for finer cuts. Here, we use 2D clearing to drill the entrance and exit holes in the device's top channel. The example device cuts are described in Figure 4. Fabrication steps in Fusion are described below for the example part. The Fusion file is available in Supplementary Materials. All steps utilize the tool settings from Table 4 unless otherwise noted.

**Table 4.** Tools and their parameters to fabricate example device

End mill	Manufacturer	Cutter parameters	Cutting data
500 μm square, 2-flute	Harvey Tool # 741411	Diameter: 0.5 mm Shaft diameter: 3 mm Overall length: 38 mm Length below holder: 24 mm Shoulder length: 2 mm Flute length (length of cut): 1.5 mm	Spindle speed: 10,000 rpm Surface speed: 51.53 ft/min Cutting feed rate: 1.005 in/min Lead-in/out: 0.75 in/min Transition/ramp: 0.5 in/min Ramp angle: 2 degrees Plunge: 0.25 in/min Stepdown: 0.25 mm Stepover: 0.125 mm Coolant: Disabled
200 μm ball, 2-flute	Performance Micro Tool #TR-2–0080-BN	Diameter: 0.2 mm Shaft diameter: 0.2 mm Overall length: 38 mm Length below holder: 24 mm Shoulder length: 0.75 mm Flute length (length of cut): 0.61 mm	Spindle speed: 30,000 rpm Surface speed: 62.83 ft/min Cutting feed rate: 0.483 in/min Lead-in/out: 0.36 in/min Transition/ramp: 0.24 in/min Ramp angle: 2 degrees Plunge: 0.12 in/min Stepdown: 0.1 mm Stepover: 0.05 mm Coolant: Disabled

**End mill steps to fabricate the example device:**

**Tool 1:** 500 μm square, 2-flute end mill (simulation time: hh:mm:ss)

1. (01:02:28) 2D Pocket on the bottom channel faces with 1 finishing stepdown at 0.1 mm

2. (00:17:04) Bores for all 0.5 mm diameter entry/exit holes on the top channel

**Tool 2:** 200 μm ball, 2-flute end mill (simulation time: hh:mm:ss)

1. (00:33:57) 2D Pocket on the top channel to remove material for the facing step

2. (07:56:15) 3D Parallel facing on top channels with 0.02 mm maximum stepdown and stepover to finish

3. (00:23:12) 2D Circular cut for 0.5 mm diameter alignment holes.

**Total time ~10.5 h**

*Note: Our instrument’s maximum spindle speed is 60k and maximum feed rate is 22.4 in/min. These values can be obtained for any machine from its manual or by contacting the mill’s manufacturer. It is advisable to avoid using values close to the machine’s maximum capacity unless required. Tool path generation must balance speed with machining capabilities and tool wear. An optimal toolpath generates minimal burrs, does not break tools, and does not wear tools too quickly. Tool wear is apparent when chips and burrs become common, and tool breakage is likely. Although we do not use quantitative metrics to evaluate wear, we avoid using tools for more than 12 h, especially for small-diameter end mills (<1 mm).*

**C. Negative master mold fabrication via micro mill**

**Before you begin:** Operating a micro mill requires familiarity with the equipment. Prior to our step-by-step operation protocol, we describe the machine’s parts and operating principles. Homing (spatial calibration) and probe depth determination are typically performed automatically using pre-programmed algorithms, but a foundational understanding of their purpose and mechanics is essential to proper use of these features.

**Figure 5.**
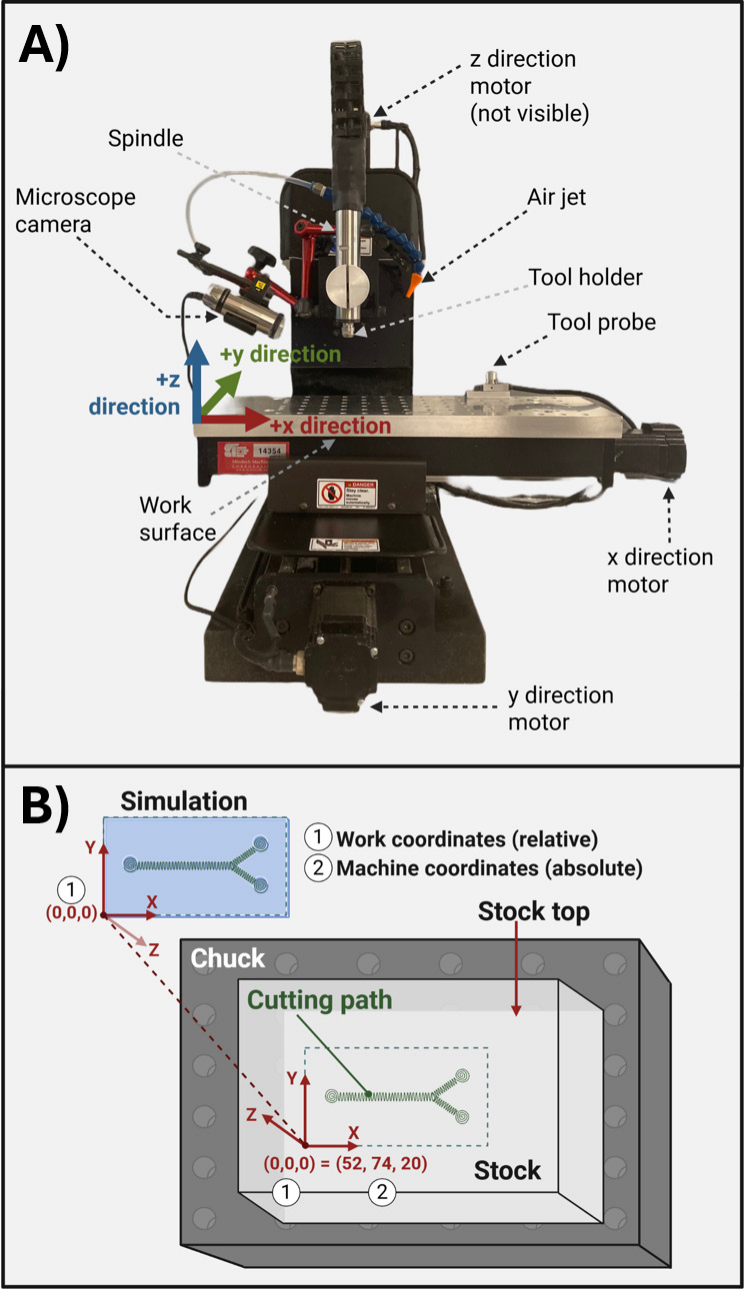
Micro mill instrument parts and coordinate geometry. (A) Micro mill machine parts with labels. (B) Stock mounted on a chuck with parts labeled. (1) and (2) represent the work and machine coordinates, respectively.

CNC machines operate by orienting the tool tip in space relative to defined reference locations. These include the home origin, the work origin, and the tool probe. Prior to cutting a part, all three of these reference points must be established. We summarize the definition of these reference surfaces in Table 5. The workbench moves in the X and Y planes, while the spindle moves in the Z plane (Figure 5). Each of these motions is enabled by stepper motors connected to a controller that is programmed using G-code that is interpreted by a software program, which, for this example, is Mach3.

The first absolute reference that is set is the home origin (Figure 5). This origin is the absolute reference against which other origins are set. This point is the limit of the X, Y, and Z axes of movement that the workbench and spindle, respectively, can achieve. The homing probes are physical sensors located at the upper X, Y, and Z limits of the machine’s range of movement. Importantly, this position is irrespective of workbench size. When the user initiates homing in the Mach3 program, the workbench and then the spindle slowly move toward these maximum X, Y, and then Z limits one at a time, until the respective probes are triggered, thus establishing the range-of-motion maximum position for each axis.

**Figure 6.**
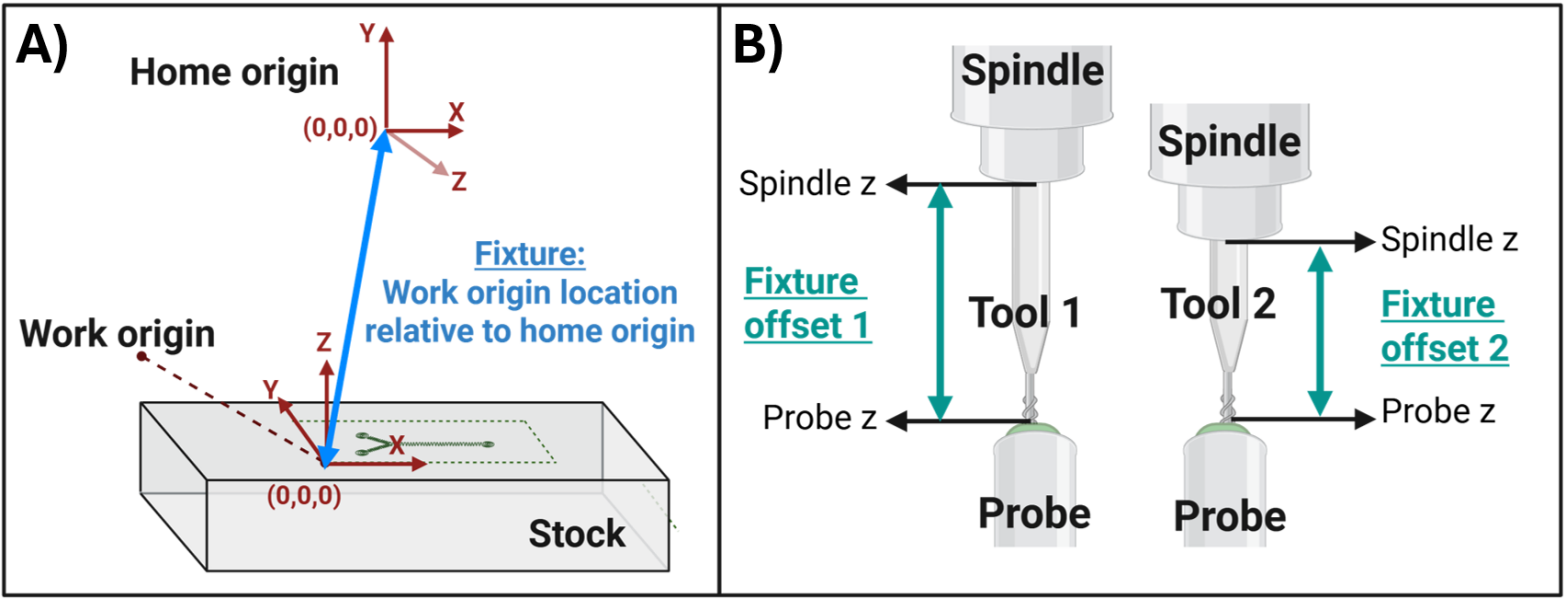
Defining the tool fixture. (A) A fixture is the set of machine coordinates, relative to the home origin, that defines the absolute location of the work origin. (B) The fixture offset is the distance between the top of the probe and the bottom of the spindle nose. This distance is calculated every time a new tool is installed to ensure that the tooltip correctly contacts the work origin despite tool height variation.

Next, a relative origin is defined, called the “Work coordinates.” This origin is placed at the cutting surface and represents the origin that has been defined in the tool path simulation (Figure 5B, Figure 6A). Work coordinates are set manually before every run because stock materials vary in height, and a precise definition of the stock surface is critical to proper machining. The distance between the work coordinates and home coordinates is referred to as a “fixture” (Figure 6A). The “fixture offset” is the distance between the spindle nose and the tooltip (Figure 6B). This distance varies depending on the tool and needs recalculation every time a new tool is installed.

**Table 5.** Terminology for micro mill operation

Parameter	Feed rate
Home origin	This (0,0,0) origin represents the maximum range of the spindle in the X, Y, and Z dimensions. The home origin is the only set of absolute coordinates and serves as the reference for relative coordinates that are defined later, such as the work coordinates.
Machine coordinates	These coordinates are relative to the Home origin and define the spindle’s absolute location.
Work origin	The work origin is a (0,0,0) position that is set to match the origin that was defined in the tool path simulation (Figure 5). The difference between the work origin and home origin is called the “Fixture.”
Fixture	A set of (x,y,z) machine coordinates (i.e., coordinates relative to the home origin) that defines a work origin. Fixtures are used to store the work origin so that it is consistent across tool changes. See Figure 6.
Probe	This is a Mach3 command that tells the machine to touch the calibration probe. The machine automatically lowers the tool tip slowly until the probe is contacted. The probe height and spindle height are then defined, which allows the machine to create a value called the “Fixture offset.” This value is applied to the fixture when tool changes occur so that the work coordinates can be reached with the new tool tip despite changes in the height of the tool relative to the spindle.
Home + Probe	This makes the machine go to Home position and then Probe. That way, the machine knows where it is in space (Home) and where the tool tip is (Probe). You only need to do this at the beginning when you have just turned on the machine.
Spindle	This is the command to turn on the tool spinning.
Jog	Term for moving the machine in x, y, z space.
G-code	The code output by simulation software, such as Fusion, for the interpretation of simulated cutting paths by the CNC milling machine.

**Maintenance and Safety**

1. Check that there is a first aid kit under the micro mill.

2. Do not touch the machine when it is running.

3. Beware of pinch points on the y-axis rail.

4. Use the Esc key to stop the machine in emergencies.

5. Wear eye protection if the door is open or if the machine is running.

6. Wear ear protection if metal is being machined and if the machine is very loud.

7. Vacuum/air gun/brush off debris after the process is complete to keep the work area clear.

To begin this step-by-step, you should already have:

- A G-code (.tap file) on a USB drive.
- An acrylic stock cut to the right size based on your Fusion simulation.
- All of the end mills that are required to cut the part.

1. Cut the appropriately sized stock piece using a band saw.

*Note: Make sure you have at least 5 mm × 5 mm × 5 mm extra stock to prevent the tool from running into the metal chuck or running off the stock. If you are facing the machine, the +x direction is to the right, +y is away from you, and +z is up (Figure 5B). Position your stock so that the longer side is in the x direction. Check Fusion and verify that you have set the origin at the top of the stock in the bottom-left corner. Make sure this matches the way you put your stock in.*

2. Turn on the machine control box.

3. Turn on the spindle control box.

4. Turn on the computer. Wait at least 2 min after the computer boots to open Mach3. Not waiting appears to cause bugs sometimes.

5. Mount your stock to the chuck: Apply double-sided tape to the bottom of the stock piece and affix it to the chuck. Ensure that it does not move if pushed.

6. Open the Mach3 software.

7. Hit Reset (it will be blinking).

8. Move the machine upward to a height where you can install a tool.

9. Install your first tool and ensure it is secure.

10. Open the camera software and adjust the position of the camera so that you can see the tool tip clearly on the computer.

11. Position the air hose to aim directly at the end of the tool to clear debris during cutting.

12. Click *Home and probe* to calibrate the end mill and let the machine identify the tip of the end mill.

13. Using the Jog tab, navigate your tool to the bottom-left corner at the top of the stock, but do not touch the top of the stock yet.

a. Get to a position similar to where you set the origin in Fusion. You need to tell the machine where your origin is. However, make sure to leave a bit of stock around your origin because you do not want to break the tool.

14. When you are satisfied with your XY position, you can slowly lower in the Z direction. When you get close to the surface, turn on the spindle and use the “step jog” function with a small step size (1–0.1 μm depending on the z resolution desired) to slowly approach the surface. When the spindle touches the surface, there is usually a sound change, so you can listen for that. But also use the camera, because sometimes with the smaller tools you cannot hear any change.

15. When you have reached the stock top with the tool:

a. Go back to *Control* and turn off the spindle.

b. On the top left, locate *Work Coordinates* and click *Zero All* to set your work coordinates (Figure 6A).

16. Now you can start running your G code:

a. From your flash drive, move your G code to the desktop.

**Caution:** Never operate a G-code directly from the flash drive. Always move the file to the computer’s hard drive.

b. On Mach3, load the G code.

c. Click *Spindle* to start the spindle. The code is “supposed” to start the spindle automatically, but this often does not work, leading to tool breakage unless the spindle is started manually.

d. Click *Cycle Start* twice to begin running your G code.

17. Tool changes: if you have multiple tools in your program from Fusion, the machine will stop when you have to do a tool change. When the machine stops:

a. Wait for the spindle to stop completely.

b. Remove the old tool and place the new tool.

c. Probe the machine to tell it where the new tool tip is.

d. Click *Cycle Start* to continue running the code where you left off.

**Caution:** Do NOT click *Home* or *Home and Probe* during a tool change. Homing should only be performed prior to a cut. Homing during a cut may result in very minor deviations in the Home position, which may interfere with the precise overlapping of features during a cut with multiple tools.

18. Finishing up: When your G-code is done running, the spindle will turn off, and the machine will move the end mill away from the stock.

a. Verify that the spindle is off and then remove your part.

b. Vacuum out the machining area to remove debris from the cut.

19. Turn everything off:

a. Turn off both air sources.

b. Close all software and turn off all machine components and the computer.

**Part II: PDMS device fabrication with the micro-milled part**

**A. Fabrication of the positive master molds**

**Figure 7.**
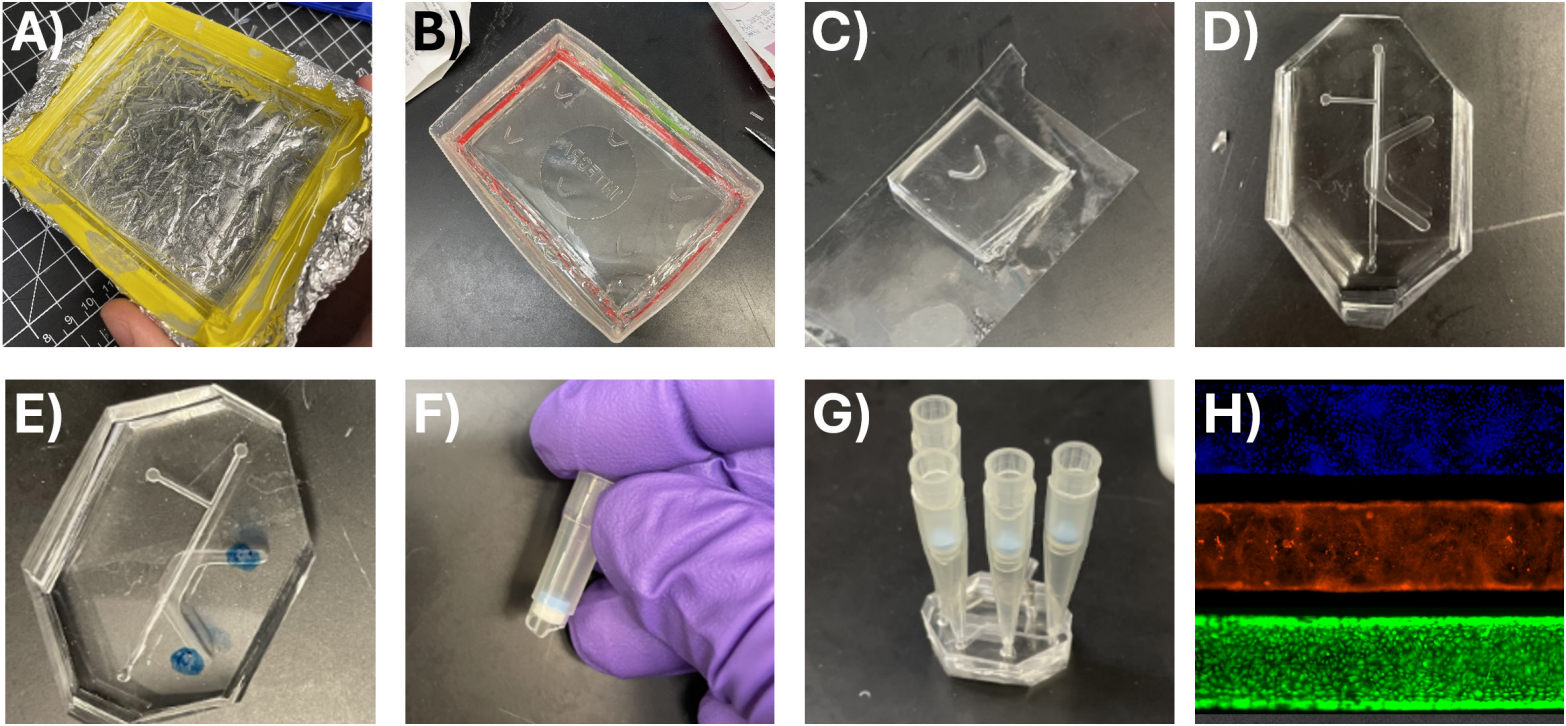
Device casting and assembly. (A) Acrylic negative master mold of the top channel lined by lab tape to contain PDMS for casting the PDMS-positive master mold. (B) PDMS-positive mold of the base channel contained in the lid of a tip box. (C) Base channel stored on clear scotch masking tape to protect from dust. (D) Top and bottom channels cut out and aligned with the corners cut off. (E) Top channel marked for cutting holes with biopsy punch. (F) Top of P200 with filter for use in cell culture. (G) Assembled device with cutoff pipette tips serving as reservoirs for the top and bottom channel media. (H) Cell culture in the microfluidic top channel demonstrates confluence of primary human small airway epithelium. Stains from top to bottom: DAPI (nucleus), CellMask Orange (plasma membrane), Calcein AM (live cell marker).

1. Prepare the acrylic top and bottom molds for casting by lining the casting area with tape or placing the acrylic mold in another container to contain the PDMS (Figure 7A).

2. Measure PDMS and crosslinker into a plastic container at a 5:1 ratio of PDMS to crosslinker.

3. Mix in the orbital mixer at 2,200 rpm for 3 min. Alternatively, manually stir for at least 5 min until well-mixed.

4. When mixing is complete, place the master mold on a gram scale and tare to 0. Pour the well-mixed PDMS plus crosslinker into the casting area on the acrylic mold until the PDMS layer is the desired thickness. We usually aim for ~0.5 cm thickness; this is enough for the mold to be sturdy but not so much that it wastes material or distorts when curing and demolding. Note the mass of PDMS required to reach this thickness so it can be matched in future casts.

5. Place the mold with PDMS flat inside a vacuum desiccator for 30 min to remove air bubbles. If bubbles remain on the surface after 30 min, use compressed air or a Pasteur pipette to blow air above the bubbles and pop them. If bubbles remain, continue under vacuum until all bubbles are removed.

6. Place the master mold in the 65 °C oven for 2 h.

7. Remove the PDMS cast from the acrylic mold. Bake the PDMS master mold, laying flat in a 120 °C oven overnight (~16 h).

8. Remove the PDMS master mold from the heat after curing.

**Caution:** Do not bake the acrylic milled negative mold at 120 °C. Only bake for 2 h at 65 °C and then remove the PDMS mold from the acrylic for the overnight 120 °C bake.

**Pause point:** Acrylic molds can be reused indefinitely. PDMS master molds can be stored and reused for several months; in our hands, they survive at least 10 use cycles before some brittleness or stickiness may prevent continued use.

**B. Cast the microfluidic components**

1. Prepare the top and bottom channel PDMS-positive master molds for casting by placing them in a larger container or lining the edges with tape as in Figure 7B.

2. Weigh PDMS and crosslinker to create a 10:1 mass-to-mass ratio and mix in an orbital mixer at 2,200 rpm for 3 min.

3. Place the mold plus container on a gram scale and tare to 0. Pour the well-mixed PDMS plus crosslinker into the casting area on the PDMS master mold until the liquid PDMS layer is the desired thickness. We usually aim for ~0.5 cm thickness; this is enough for the mold to be sturdy but not so much that it wastes material or makes devices too thick for adequate gas exchange during later cell culture steps. Note the mass of PDMS used for this cast so that the same amount is applied in subsequent casts for consistent device thickness across batches.

4. Place the PDMS mold horizontally in a vacuum desiccator for 30 min until all bubbles are removed.

5. Bake in a 65 °C oven, laying flat, for 2 h. Do not overbake, as this causes the devices to become brittle and less adhesive during device assembly.

6. Remove baked PDMS and mold from the oven and allow to cool at the bench until room temperature.

7. Use a straight-edge razor or a very sturdy scalpel to outline the cast PDMS area and peel it slowly and carefully off the positive master mold to avoid ripping the cast PDMS.

8. Cut the cast PDMS into individual channels for each device and store on masking tape to prevent dust collection on the inner channel and adhesive surfaces (Figure 7C).

*Note: Cast PDMS parts can be stored on masking tape for several months without issue, but after ~6 months, the PDMS seems to bond to the tape and is no longer usable.*

**C. Surface-functionalize the porous membrane**

*Note: For PDMS sandwich devices, ensuring that bonds are leak-proof during cell culture is critical. Plasma oxidation–induced chemical bonding is the most robust method for preventing leaks after devices are assembled. However, commercially available porous membranes, i.e., Transwell membranes, are comprised of polycarbonate that cannot be covalently conjugated to PDMS via plasma oxidation directly. Therefore, we must functionalize the surface of the polycarbonate membrane with (3-Aminopropyl) triethoxysilane (APTES), to allow plasma oxidation–induced covalent bonding to PDMS. This has, in our experience, led to robust leak-free bonding for long time periods in cell culture (2+ weeks) for almost all devices. Our method is adapted from one that was described by Zhang et al. [17].*

**Caution:** This protocol requires the handling and heating of APTES, which is a caustic, hazardous chemical. Wear appropriate PPE and work in a certified chemical fume hood while handling this material. Gloves and disposables that are contaminated with APTES should be collected for chemical hazard waste according to institutional guidelines. **DO NOT remove the bottle’s septum cap.** The septum cap prevents air from entering the container where it could react with APTES. Use a syringe and needle inside a fume hood to obtain APTES from the stock solution.

*Note: Up to 12 membranes can be processed at one time. More than this would require two people.*

1. Bring a glass beaker (400 mL) into the fume hood along with APTES, a 10 mL plastic syringe, and a 16G (or similar size) needle.

2. Add 190 mL of water to the glass beaker and place a magnetic stir bar in the beaker.

a. The stir bar can be any size, but we have found that a length of ~50% of the beaker’s diameter is effective.

3. Place the beaker on a magnetic stirring hotplate and turn on the stirring to ~4/10. Do not turn on the heat yet.

4. Extract 10 mL of APTES from the stock by applying the needle through the septum cap. Draw slowly to avoid creating vacuum-induced bubbles.

5. While stirring the water, slowly add APTES to the water by pushing it out of the syringe over ~20 s.

6. Cover the beaker firmly with aluminum foil to reduce evaporation.

**Caution:** NEVER heat a liquid in a completely closed container. This can cause an explosion.

*Note: In our experience, most hotplates do not accurately produce the temperature they are set to. Monitor the liquid’s temperature using a laser thermometer and make adjustments when needed to maintain a temperature between 75 and 85 °C for the duration of the reaction.*

7. Turn on the hotplate and set it to 350 °C.

8. Wait 10–20 min for the liquid to reach 80 °C. Monitor the liquid temperature using a laser thermometer.

9. When the liquid reaches the temperature, prepare the membranes for functionalization.

a. Plasma oxidize four membranes at a time (50 W for 1 min). Both sides of the membrane require functionalization, so the membrane is oxidized twice so that both sides are exposed to plasma. The side facing down does not get good exposure to the plasma.

b. While the second batch of membranes is being oxidized, use a scalpel and forceps to carefully cut the membrane two-thirds of the way around, but not entirely out of its plastic holder. Place it on a clean, dry surface until all membranes are ready. Do not stack membranes during this step.

*Notes:*

*1. Effects of plasma oxidation become less potent with time on the scale of minutes to hours, so speed is relatively important. However, a difference of 10–20 min between plasma oxidation and submersion in APTES does not seem to affect membrane adhesion and leak prevention in the devices.*

*2. We have found that completely cutting the membranes out causes them to stick to each other during the reaction, preventing effective surface functionalization. We have found that leaving the membrane entirely attached causes severe wrinkling and uneven coating.*

10. Once all 12 membranes have been plasma-oxidized twice (once for each side of the membrane), bring them into the fume hood.

11. Remove the foil from the heated beaker using forceps.

12. Use forceps to place the membranes one at a time into the 80 °C APTES mixture.

13. Start a timer for 20 min and monitor the hotplate to ensure the temperature remains within range (80 ± 5 °C).

14. When the timer is done, turn off the hotplate. Leave the solution stirring on the hot plate and wait 20 min for the liquid to cool down before handling.

15. After 20 min, use heat protection gloves (e.g., oven mitts) to carefully remove the beaker from the heat and place it on the bench.

16. Use forceps to fish each membrane out of the beaker and place it carefully on a paper towel. Try to ensure the membrane is not touching anything (except the part of the holder it is still attached to), or it will ruin that part of the membrane.

17. Once all membranes are drying, dispose of chemicals and disposables in hazardous and sharps waste according to institutional guidelines. For the APTES-contaminated beaker, rinse the beaker with water several times and dispose of this rinse in the chemical waste. After at least three water rinses, the beaker can be rinsed in the sink.

18. Membranes should be allowed to dry in the fume hood with the hood closed at least overnight (16 h).

19. Functionalized membranes can be removed from their plastic holders and stored in a cool, dry place protected from dust for at least 6 months. Once dried and cooled off, they do not bond to other plastics (e.g., Petri dish) or glass during storage.

**D. Assemble the sandwich device**

1. Cut the functionalized membranes into rectangles that are large enough to cover the top and bottom channels completely.

2. Use 1.5 mm diameter biopsy punches to create entrance and exit holes ONLY on the top channel.

3. Match each top channel to a bottom channel: remove the top and bottom PDMS channels from masking tape storage. Align them properly according to the guide markers. Then, carefully cut the corners off each corner as vertically as possible. This facilitates easier alignment later after plasma oxidation and final assembly (Figure 7D).

4. Once all devices are paired, aligned, and cut, and all membranes are cut, proceed to the next step.

5. Place 1–3 top channels, with the channel facing up, and their membranes on a flat surface, and load it into the plasma oxidizer.

a. We use four large glass slides that have been covered with lab tape so that the PDMS does not bond to them after plasma oxidation.

b. Use a small amount of lab tape or scotch tape to pin down the edge of the membrane, or it will fly away during plasma oxidation or while it is being moved.

6. Plasma-oxidize the top channel and membrane (50 W, 30 s).

7. Immediately move the top channel with the membrane to the bench. Use a forceps and a scalpel to cut the membrane off the piece of tape and carefully lay it on the top channel in its proper orientation.

a. The membrane bonds PDMS immediately on contact, so it is critical to properly align and lower the membrane to avoid wrinkling and poor placement. Improperly bonded membranes cannot be removed, so the entire bottom channel and membrane must be discarded.

b. Plasma oxidation is less potent with time, and for this step, it is critical to place the membrane within 1 min (preferably 30 s) of plasma oxidation, or the adhesion is not as effective.

8. Once the membrane is attached, plasma-oxidize the top and bottom layers with the channel side facing up (50 W, 30 s).

9. Immediately after oxidizing, align the top and bottom channels and place the top channel on top of the bottom channel. Apply mild pressure for ~5 s to ensure contact between layers.

10. Place the device in an oven at 65 °C for 20–30 min to solidify the bonding between layers.

*Note: Do not over-bake devices, as this causes brittleness and possible cracking. Do not bake at a higher temperature, because the membranes wrinkle.*

**Part III: Cell culture in the device**

*Note: In this example, we culture primary human small airway epithelial cells in the microfluidic device to create a lung-on-a-chip. These cells were cultured according to the manufacturer’s instructions; therefore, those protocols are omitted here for brevity. This protocol begins on the day that cells are 60%–80% confluent and ready for passage.*

**A. Seed and culture small airway epithelium**

1. In a sterile biosafety cabinet (BSC), use a straight razor to cut a P1000 pipette tip about halfway down. Repeat to create four cutoff tips per device.

*Note: Cut the P1000 tip at the right diameter so that the P200 filter component can sit in the P1000 tip (see Figure 7G). Most brands of pipette tips will work for this setup.*

2. In a sterile BSC, use a straight razor to cut a P200 pipette tip containing a sterile filter approximately right below the sterile filter.

3. Store these under sterile conditions indefinitely until use.

4. To begin seeding devices, make a solution of rat tail collagen type I in PBS at pH 9 on ice.

a. Obtain collagen in a biosafety cabinet and add to PBS to create a solution at 91 μg/mL. The collagen concentration is lot-dependent and is printed on the bottle. Typically, the volume is close to 26 μL per milliliter of PBS.

b. Use 1 M sodium hydroxide to adjust the pH to 9. This helps to prevent gelation during the collagen coating step.

c. Filter-sterilize the collagen solution through a 0.22 μm syringe filter and store at 4 °C for up to one month.

5. In a sterile biosafety cabinet, bring in microfluidic devices on a sterile Petri dish.

6. Use scotch tape to clean debris and dust off the outside surfaces of the device.

7. Pipette 70% ethanol into the top and bottom channels of the device and let sit for 30 s. This sterilizes the device to prepare for cell culture.

8. Aspirate 70% ethanol from the devices and rinse the top and bottom channels with 3 mL of PBS to clear ethanol.

9. Aspirate PBS from the top and bottom channels so that they are now air-filled.

10. When all devices are sterilized and rinsed, use a P100 to pipette ~13 μL of collagen solution into the top channel. Surface tension keeps the solution from bleeding into the bottom channel.

11. Once all devices are filled, incubate them for 90 min in a cell culture incubator (37 °C, 95% humidity, 5% CO_2_).

12. About 15 min prior to reaching the 90-min incubation, begin passaging airway epithelial cells in preparation for seeding.

*Note: Passaging is conducted according to the manufacturer’s instructions. For more information, see the product page for Lonza Biosciences catalog CC-2547, SAEC: Human small airway epithelial cells (https://bioscience.lonza.com/lonza_bs/US/en/Primary-and-Stem-Cells/p/000000000000185029/SAEC-%E2%80%93-Human-Small-Airway-Epithelial-Cells). Scroll down to locate the document entitled “Instructions - Airway Epithelial Cell Systems.”*

13. When cells are passaged and resuspended in cell culture media, count cells and adjust their concentration to 3 million cells/mL.

14. Prepare the microfluidic devices for seeding.

a. Insert P1000 cutoff pipette tips in each channel entrance (except the T channel).

*Note: In this example, the device contains a T-channel that is not used for cell culture. For this procedure, this channel is plugged with a sterile PDMS plug to prevent cell seeding in this area and concentrate medium flow in the main channel.*

15. Use a P200 pipette to add 200 μL of cell culture media to the bottom channel by pipetting media into the cutoff pipette tip reservoir.

16. When collagen incubation has reached 90 min, displace the collagen solution with 100 μL of cell culture media. Then, aspirate the collagen and media from the top channel until there is only enough media remaining to cover the channel, but none in the reservoirs.

17. Seed 50 μL of cells (at 3 million cells/mL) into the top channel by pipetting into the reservoir furthest from the T-channel. The cells will flow into the T channel if seeded from the other side, which is undesirable.

18. Slowly aspirate 20–30 μL of medium out of the opposite top channel reservoir closer to the T joint to gently pull cells into the channel.

19. Incubate devices in a cell culture incubator for 2 h to allow cell attachment.

*Note: If bubbles appear in the device channels, the incubator’s moisture could be low. Alternatively, there could be a leak. Inspect the Petri dish that is holding the devices to see if liquid has accumulated. This indicates leaking.*

20. After 2 h, gently add 100 μL to the inlet top channel, wait 30 s, then gently remove 100 μL from the outlet. Repeat two more times. This clears unattached cells that would otherwise die and/or steal nutrients from attached cells.

21. Inspect channels to ensure no large cell aggregates remain in the channel or in either channel entrance. These can interfere with the cell culture of attached cells.

22. Fill the pipette tip reservoirs until all four media levels reach a height of about 1 inch. It is important that the media height in the top and bottom channels is approximately equal to prevent pressure-driven flow across the porous membrane during cell culture.

23. Place the Petri dish containing devices on a rocker plate so that the device channel is shifted from one side to the other to create flow from one reservoir to the other and back. This enables adequate medium exposure to cells without the requirement for pumps, tubing, and other advanced equipment.

24. Completely change the top and bottom channel media every 24 h until the experiment. We have cultured for up to 2 weeks using this method.

## Data analysis

An effective design and fabrication will produce devices that do not leak and produce a confluent, live epithelial layer as in Figure 7H. The fabricated device is further applied to collect data such as epithelial barrier permeability and cellular response to various insults and experimental conditions. Detailed description of these outcomes is outside the scope of the present work, but interested readers are referred elsewhere [18–20].

The quality of your part can be evaluated through visual inspection using a stereoscope. A quality cut should be free of burrs and have a smooth surface with no burring, inadvertent sloping of vertical or other sheer features, and minimal grooving. Detailed descriptions of visual features indicative of suboptimal machining are described in excellent references [20,21]. The relationship between spindle speed and surface roughness for relevant substrates, including plastics, has been characterized. Micro milling generally produces surface roughness from 0.42 to 1.5 μm on plastic surfaces, and this appears compatible with cell culture viability of at least 95% [2,23]. Also, it should be noted that PDMS can shrink during casting. Several references have addressed this issue [24,25].

Additionally, many quantitative micro milling studies employ white-light interferometry, scanning electron microscopy, confocal microscopy, and atomic force microscopy to characterize microscale feature shapes, dimensions, and surface roughness. Metrics for successful surface finish will vary depending on the application. A recent preprint provides a detailed characterization of micro-milled microfluidic channel features for application to cell culture studies using different instruments, offering a benchmark for success in biological applications [26]. For more information, we refer interested readers to excellent literature [27–31].

## Validation of protocol

This protocol has been used and validated in the following research article:

- Viola et al. [8]. Liquid plug propagation in computer-controlled microfluidic airway-on-a-chip with semi-circular microchannels. *Lab Chip*.

## General notes and troubleshooting

**General notes**

1. A two-flute is preferred for the micro mill because it is easier to clear chips (leftover material from cutting).

2. Helix angle should be <30° for micro milling.

3. A drill bit (rather than end mill) is required for holes with a depth-to-diameter ratio greater than 3:1.

4. Four flute end mills have lower surface roughness but poorer chip removal.

5. Surface roughness is directly proportional to the feed rate (high feed rate, high roughness).

6. Burrs are common in ductile plastics, which may necessitate lower chip loads.

7. Dull tools, especially with low chip load (high spindle speed or low feed rate), cause high friction and heat. This increases material ductility and can result in burrs.

a. Do not use feed rates too low or spindle speeds too high, especially with dull end mills.

**Troubleshooting**

Optimization of micromachined cuts is not an exact science and requires empirical observation of the finish quality and tool wear rate. Therefore, it is often an iterative process that attempts to minimize undesirable features. Common undesirable outcomes of CNC machining include burring, rough surface finish, and broken tool tips. These can result from any number of variables, including suboptimal cutting parameters, tool size and tip shape, material selection, or inadequate lubrication. However, the most common culprit is suboptimal cutting parameters that result in high chip load and overheating of the tool tip.

**Table:** 

Protocol step	Problem	Solution(s)
Part I, step A6 SolidWorks model	Features are distorted or broken if other unrelated elements in the model are changed	Delete the broken feature. The sketch underlying the feature will be preserved. Enter the sketch and identify which lines, points, or other elements are becoming skewed. **Fully define the sketch:** It is possible that the sketch is not fully defined, in which case, line(s) in the sketch appear blue rather than black, and changing the model in seemingly unrelated ways could cause the sketch to deform. Create mating relationships to reference geometry to fully define the sketch. **Examine mating relationships:** If the sketch is already fully defined, identify all mating relationships within sketch elements. Ensure that mating relationships are formed only between sketch elements and reference geometry (e.g., planes, lines, and points). It is possible that an inadvertent mating relationship exists between one or more of the sketch elements and another part of the model, such as a face, another sketch, or a model feature. In this case, modifying the model will cause the sketch to change so that mating relationships are preserved. We recommend fully defining sketches and using only reference geometry for these definitions so that modification of other model elements, like features, other sketches, and other parts, does not cause inadvertent sketch deformation. Ensure that all construction lines, such as spline guides, are mated fully to define splines. If necessary, create reference planes specifically for this purpose.
Part I, step B6 Tool path simulation	Long cutting times	**Suboptimal cutting path design:** Use the largest possible tool for clearing material prior to the final surface cuts. Use smaller tools for fine features where required. Ensure that unnecessary motion of the spindle is reduced in Fusion by ensuring that cuts are performed by area rather than by height. Reduce retraction heights to the minimum required. Increase spindle speed so that the feed rate can be increased. Mill the negative pattern and perform PDMS double casting to reduce the surface area that requires milling.
Part I, step B6 Tool path simulation	Ridging artifact	**Smaller stepover:** Add a finishing step to the tool path that uses a small stepover (<50% tool diameter) using a facing cut like Parallel or Scallop. Ensure that the feed and speed are low for the facing step to prevent a rough finish. **Suboptimal end mill or tool path:** Tools are available in many geometries for different types of cuts. A ball nose end mill is more suited to curved features, while a square or flat end mill is designed to produce a smooth finish on flat surfaces. Additionally, the end mill diameter should match the feature size appropriately.
Part I, step C16 Tool path design	Tool breakage, burrs, chip/substrate melting, poor surface finish, poor feature resolution	**Chip load is too high:** Reduce chip load by reducing parameters like the feed rate, spindle speed, maximum roughing stepdown, and maximum stepover to place less stress on the end mill. Alternatively, depending on the features being cut, a softer material may be required. **Spindle speed is too high:** High spindle speeds can produce vibrations that affect surface finish. Lower spindle speeds reduce this effect. **Inappropriate substrate for model design:** Some substrates, like ductile plastics, are prone to melting, which can produce an uneven surface finish in particular situations. A harder substrate or one with a higher melting point may be necessary. **Inappropriate tool for features:** Tools are available in many geometries for different types of cuts. A ball nose end mill is more suited to curved features, while a square or flat end mill is designed to produce a smooth finish on flat surfaces. Additionally, the end mill diameter should match the feature size appropriately. **Tool is dull:** A dull tool will produce artifacts like burrs, chips, and a rough surface finish. Replace the tool and note how many hours it was used to benchmark its useful life for future cuts.
Part I, step C16 Milling master mold	End mill is colliding with the stock, chuck, or workbench	**Improper origin setting in Fusion or on the mill** (Part I, steps B4–5, or Part I, steps C14–15): Review the tool path simulation (Fusion) to determine whether collisions are predicted. If not, ensure that end mill parameters are entered in Fusion as precisely as possible. Ensure that the Origin in Fusion is set to the correct location (top of stock, bottom-left corner). **Spindle is not on when it contacts the stock (Part I, step C16d):** The spindle may need to be engaged manually after tool changes and before the first cut is made. Ensure that, for helix bore cuts, the spindle is engaged with enough clearance above the stock so that it never hits the stock before turning on.
Part I, step C16 Milling master mold	Mismatched finish height	**Description:** Some tools, especially with very different shapes and sizes (5+ orders of magnitude, e.g., 100 vs. 1000 μm), can produce different finish heights even at the same fixture offset. In some cases, even the same tool can produce different finish heights for different cuts. Although this is usually a minor effect, it can be a significant issue in micro- and nano-scale applications. **Home and probe:** Ensure that homing is performed only prior to a cut and not between tool changes. Ensure probe occurs before the first cut and after every tool change. **Substrate flexion:** Minor flexing or expansion of the substrate material during milling can cause finish heights to slightly differ. A harder substrate, alternative end mill, or more aggressive stock securing method (e.g., clamps rather than tape) may be effective. Additionally, suboptimal end mill selection for the features being cut, suboptimal tool path design, and/or high feed/speed parameters can exacerbate substrate deformation by creating heat and high forces during cuts. **Tool path design:** One strategy to avoid this artifact is to mill features with a small stock to leave (0.5 mm or less). Then, perform the final finishing step with one tool using a uniform parallel path that covers the entire device area. **Casting method:** Some designs requiring a ductile plastic substrate that suffer from mismatched finish heights may be amenable to PDMS double casting, which allows the design to be milled as a negative. This avoids the requirement to mill the space surrounding the features, which is where uneven finishing height may cause problems with device adhesion or channels leaking.
Part II, step B7 Device PDMS casting	Brittle PDMS with features easily damaged during the removal of PDMS-negative device	**Peeling cast PDMS off the mold too quickly**: The PDMS molds can be separated from cast PDMS devices, but one must use careful and slow movements to avoid ripping the cast PDMS or damaging the mold. Sometimes, waiting for the mold to completely cool before removing the cast PDMS layer can improve separation. **Overbaked mold:** PDMS-positive molds are only reusable to a certain degree because repeated baking eventually causes excessive brittleness. When features are becoming damaged by continued use of the PDMS master mold, make a new one.
Part III, step A23 Cell culture	Devices are leaking during cell culture	Leaking may be readily apparent via microscopy, but may only be apparent by the appearance of large bubbles in the microfluidic channels. **Improper device assembly:** Devices are most prone to leakage due to improper bonding of the top layer, membrane, and bottom layer. Ensure that each layer is appropriately exposed to oxygen plasma, facing up, before bonding. Bake assembled devices to solidify bonds after assembly.
Part III, step A23 Cell culture	Cells are not viable, or not reaching confluence	Dead cell aggregates: Ensure that cell aggregates are not trapped in entry or exit channels, as this may deplete culture media or become toxic to cells in the microfluidic channel. Media flow: Ensure that the rocker is moving to a high enough angle for full exchange of media across the channel. Provide enough media in both the top and bottom reservoirs to ensure adequate support to cells. Change media at least once every 24 h. Contamination: Use media with penicillin and streptomycin. Sterilize devices before seeding cells. Ensure the devices are not leaking or becoming contaminated. Conduct mycoplasma testing.

## Supplementary information

The following supporting information can be downloaded here:

1. MicroMill Feed Speed Calculations

2. SolidWorks files

3. Example Fusion and G code
